# Effect of pneumatic vitreolysis in management of patients with symptomatic focal vitreomacular traction

**DOI:** 10.1186/s40942-022-00376-2

**Published:** 2022-03-28

**Authors:** Ahmed N. Fouad, Iman M. Eissa, Ghada A. Nassar, Mahmoud Leila, Adel M. Fathy

**Affiliations:** 1 Retina Department, Research Institute of Ophthalmology, 2 Al Ahram st., Giza, Egypt; 2 Department of Ophthalmology, Faculty of Medicine, Cairo University, Cairo, Egypt

**Keywords:** Pneumatic vitreolysis, Intravitreal injection of SF6, Focal vitreomacular traction

## Abstract

**Purpose:**

To evaluate the efficacy of single intravitreal injection of an expansile concentration of sulphur hexafluoride gas (SF6) in treating patients with symptomatic focal vitreomacular traction (VMT) documented by spectral domain optical coherence tomography (SD-OCT) preoperatively.

**Methods:**

This is a prospective interventional case series including 30 eyes of 29 patients with symptomatic focal VMT evident on SD-OCT. Pre-operatively, mean best corrected visual acuity (BCVA) was 20/125 (range 20/400–20/40). Mean central foveal thickness (CFT) was 382 μm (range 149–576 μm; SD ± 91.88). All eyes received single intravitreal injection of 0.3 mL of 100% SF6 gas. Postoperatively, we performed SD-OCT at one week, one month, and three months for all eyes. Primary outcome measure was release of VMT. Secondary outcome measures were changes in postoperative BCVA andCFT.

**Results:**

Overall, VMT release occurred in 24 of 30 eyes by the final follow-up visit (80% final release rate); furthermore, 76.9% of eyes with diabetic maculopathy and 25% of eyes with concurrent epiretinal membrane (ERM) had successful VMT release. VMT release was documented on SD-OCT at an average of 3 weeks (range, 1–12 weeks). The rate of release in phakic eyes was 90% (18 of 20 eyes) versus 60% in pseudophakic eyes (6 of 10 eyes). One patient developed a retinal break at upper nasal retina after two weeks of injection.

**Conclusion:**

Pneumatic vitreolysis (PVL) with limited face-down position is a viable option for treating focal VMT with few adverse events. Further studies are needed to evaluate its indications, benefits, and risks.

## Introduction

Vitreoretinal interface disorders refer to a spectrum of pathologic interactions between the posterior hyaloid and the underlying retinal surface, ranging from innocuous attachment to substantial disruption of retinal integrity. Vitreomacular traction (VMT) is defined as posterior vitreomacular attachment with tractional distortion of the perifoveal architecture inducing visual disturbance. VMT can occur in isolation, or in conjunction with comorbid macular conditions, as macular hole, macular edema, and epiretinal membrane (ERM) [1]. The prevalence of isolated idiopathic VMT is approximately 0.6 per 100 000 of the general population [2]. Histopathologic examination of VMT specimens demonstrates a variety of cell types such as astrocytes, myofibroblasts and fibrocytes. These glial cells contribute to the contractile forces in VMT [3]. VMT can be classified as focal (≤1500µm) or broad (>1500µm) depending on the diameter of vitreous attachment and as concurrent or isolated based on morphologic finding on OCT images. Vitreomacular adhesion (VMA) can be divided into 2 shapes according to the pattern of adhesion: V-shaped and J-shaped, the first pattern is associated with better surgical outcomes than the latter [4–6]. Although VMT is typically treated with pars plana vitrectomy (PPV) or intravitreal Ocriplasmin injection, these procedures can be invasive, require capital costs and surgical expertise as in PPV and the success rate is lower as in Ocriplasmin injection [7, 8]. Another treatment modality for VMT is pneumatic vitreolysis (PVL) with an intravitreal injection of an expansile concentration of gas bubble, potentially avoiding the need for vitrectomy or enzymatic vitreolysis. In addition, intravitreal gas injection may be a safer procedure compared to the more invasive PPV [9, 10]. In the present study, we evaluated the efficacy of intravitreal injection of an expansile concentration of sulfur hexafluoride (SF6) gas for the treatment of symptomatic focal VMT syndrome.

## Patients and methods

This is a prospective interventional non-comparative study including patients with isolated focal VMT (≤1500µm) treated with a single injection of an expansile concentration of SF6. The study was performed at the Research Institute of Ophthalmology (RIO), Giza, Egypt. The scientific committee of RIO and of the Ophthalmology department, Faculty of Medicine, Cairo University approved the study. The study followed the tenets of the Helsinki declaration 2013. The study required that all patients signed an informed consent prior to enrollment including publication of data without revealing the patient’s identity. Inclusion criteria were patients older than 40 years, maximum BCVA of 20/63 or metamorphopsia and had to have the following OCT criteria: evidence of perifoveal vitreous cortex detachment from the retinal surface, macular attachment of the vitreous cortex within a 3-mm radius of the fovea, association of attachment with distortion of the foveal surface, intraretinal structural changes, elevation of the fovea above the retinal pigment epithelium (RPE), or a combination of the above. Patients with concurrent focal VMT associated with ERM, non-proliferative diabetic retinopathy with or without diabetic macular edema (DME), retinal vein occlusion and age related macular degeneration (AMD) were also included in the study. Patients with focal VMT who had proliferative diabetic eye disease, optic nerve atrophy, glaucoma, previous retinal breaks or retinal detachment, dense cataract or corneal opacity preventing OCT measurements and patients who had broad VMT (>1500µm) were excluded from the study. The primary outcome measure was release of VMT on SD-OCT. Secondary outcome measures included changes in best corrected visual acuity (BCVA) and central foveal thickness (CFT) through the three months that followed treatment. Patients with concurrent pathology with focal VMT did not receive additional treatment other than PVL throughout the end of follow-up period. BCVA was assessed using Snellen notation and converted to decimal notation to perform statistical analysis. All patients had complete ophthalmic examination including slit-lamp examination of the anterior segment, intraocular pressure (IOP) assessment using Goldmann applanation tonometer (GAT), dilated fundus examination by slit lamp biomicroscopy and indirect ophthalmoscopy to exclude peripheral retinal breaks. OCT imaging was performed using Heidelberg Spectralis OCT (Spec-TR-04442, Germany) for the measurement of central foveal thickness (CFT) and horizontal vitreomacular adhesion (HVMA).

### Operative technique

All cases were done by the same surgeon (AN) in RIO operative theatre. Surgical procedure consisted of eye sterilization and draping, topical 0.5% proparacaine application, and sterile prepping. Prophylactic paracentesis was performed using an insulin syringe via the limbus to remove 0.1–0.2 ml of the aqueous. Afterwards, a 0.3 ml of 100% SF6 gas was injected using an insulin syringe through the pars plana into the vitreous cavity. The IOP was assessed digitally and the central retinal arterial perfusion was checked by indirect ophthalmoscopy. All treated patients were asked to avoid supine position, until resolution of the intraocular gas to avoid pupillary block and cataract formation. All Patients were instructed to adopt a “drinking bird” posture in which they pointed their nose directly toward the ground for a 10 s interval at least 4 times per hour. Post-operative treatment regimen for all patients included topical broad spectrum antibiotic and Beta-adrenergic blocker eye drops.

### Postoperative evaluation

All patients were examined for release of VMT in the first week, one month and three months postoperatively. At each follow-up visit, BCVA was measured, OCT images were obtained through a dilated pupil to measure central foveal thickness (CFT) and HVMA. Complications were recorded and managed.

### Statistical analysis

Data were analyzed using the Statistical Package for Social Science (IBM SPSS) version 23. Parametric quantitative data were presented as mean, standard deviations and ranges; whereas non parametric data were presented as median. Qualitative variables were presented as number and percentages. The comparison between groups regarding qualitative data was done by using Chi-square test and/or Fisher exact test when the expected count in any cell was found to be less than 5. The comparison between two groups regarding quantitative data and parametric distribution was done by using Independent *t*-test. Mann–Whitney test was used for non-parametric distribution. The comparison between more than two paired groups regarding quantitative data and parametric distribution was done by using repeated measures ANOVA test and by Friedman test for non-parametric distribution. Spearman correlation coefficients were used to assess the correlation between two quantitative parameters of the same group. *P* < 0.05 was considered significant and < 0.01was considered highly significant.

## Results

**Fig. 1:**
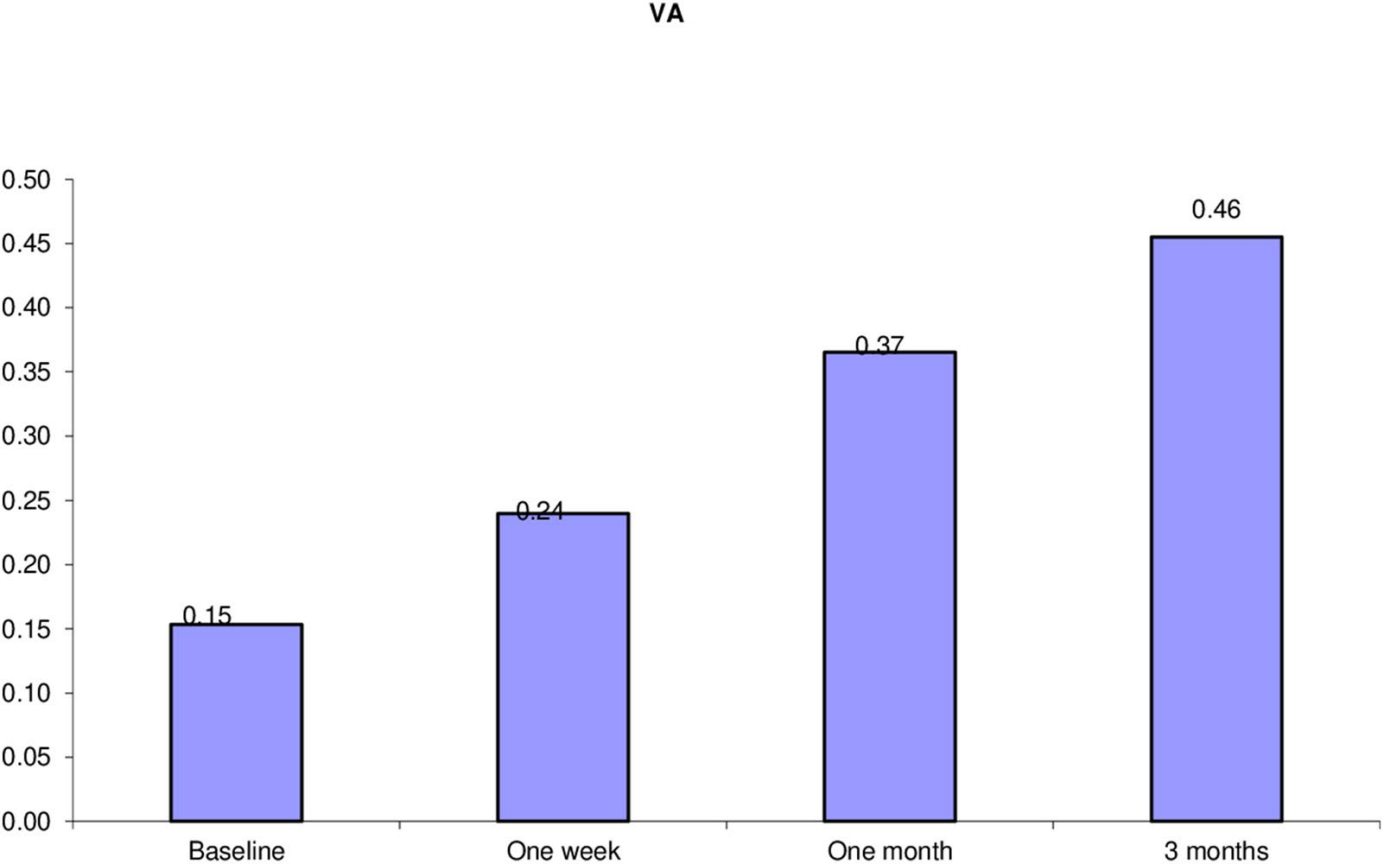
Mean BCVA over the follow up period shows gradual increase over time

**Fig. 2:**
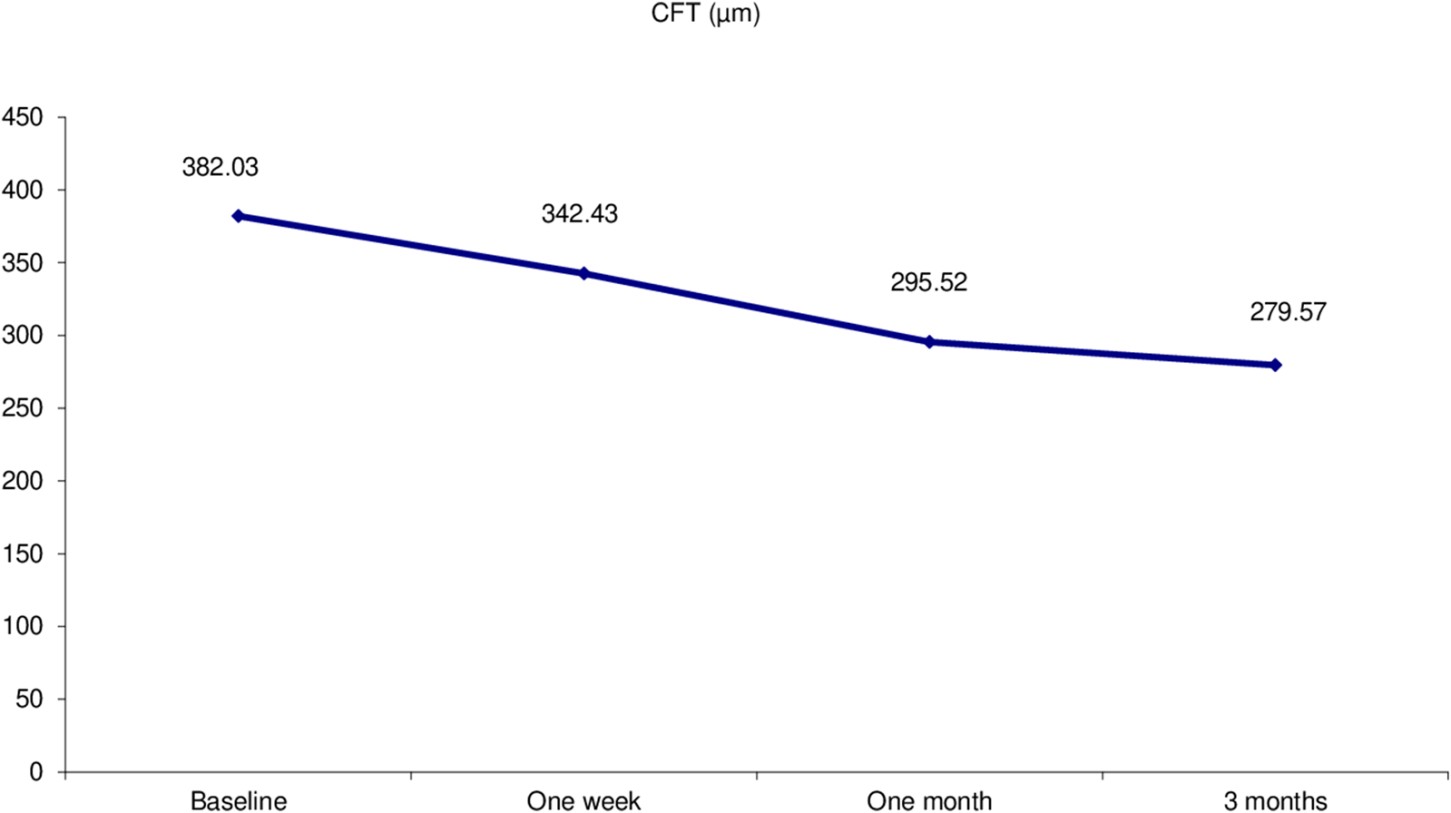
Mean CFT over the follow up period shows gradual decrease over time

**Fig. 3:**
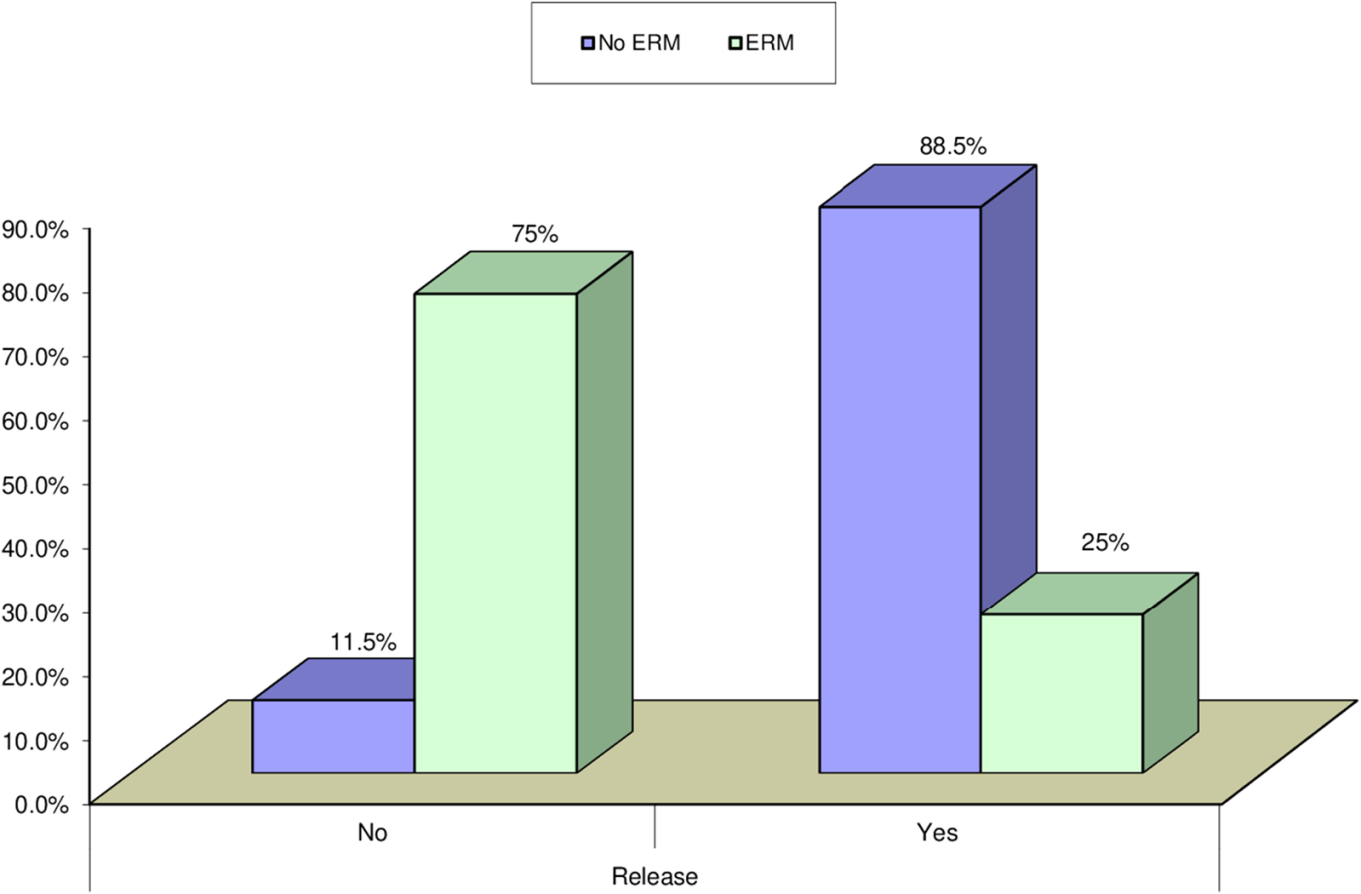
Relationship between presence of ERM and release of focal VMT, Chi-square test

**Fig. 4:**
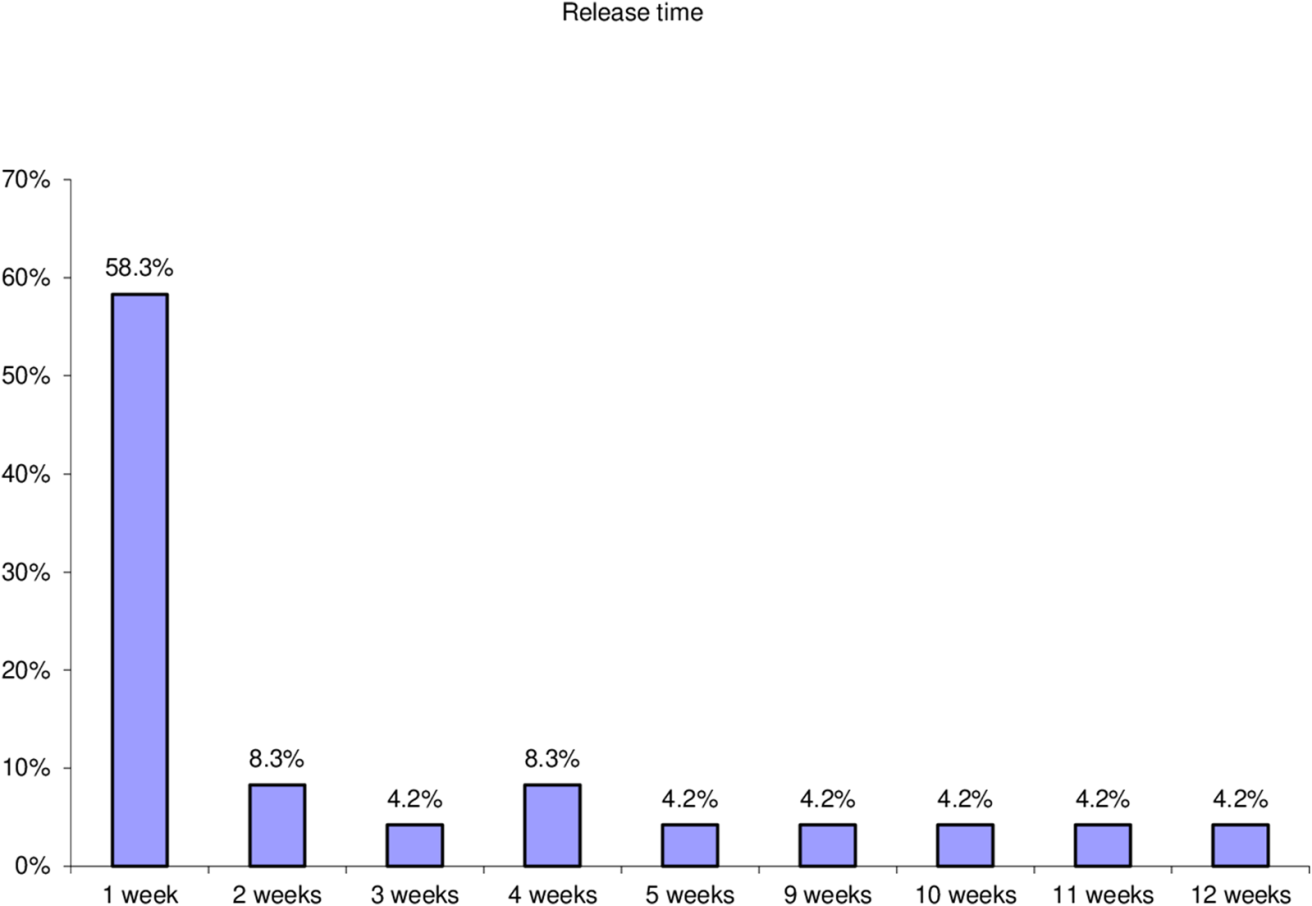
Number of eyes versus release of VMT over time

**Fig. 5:**
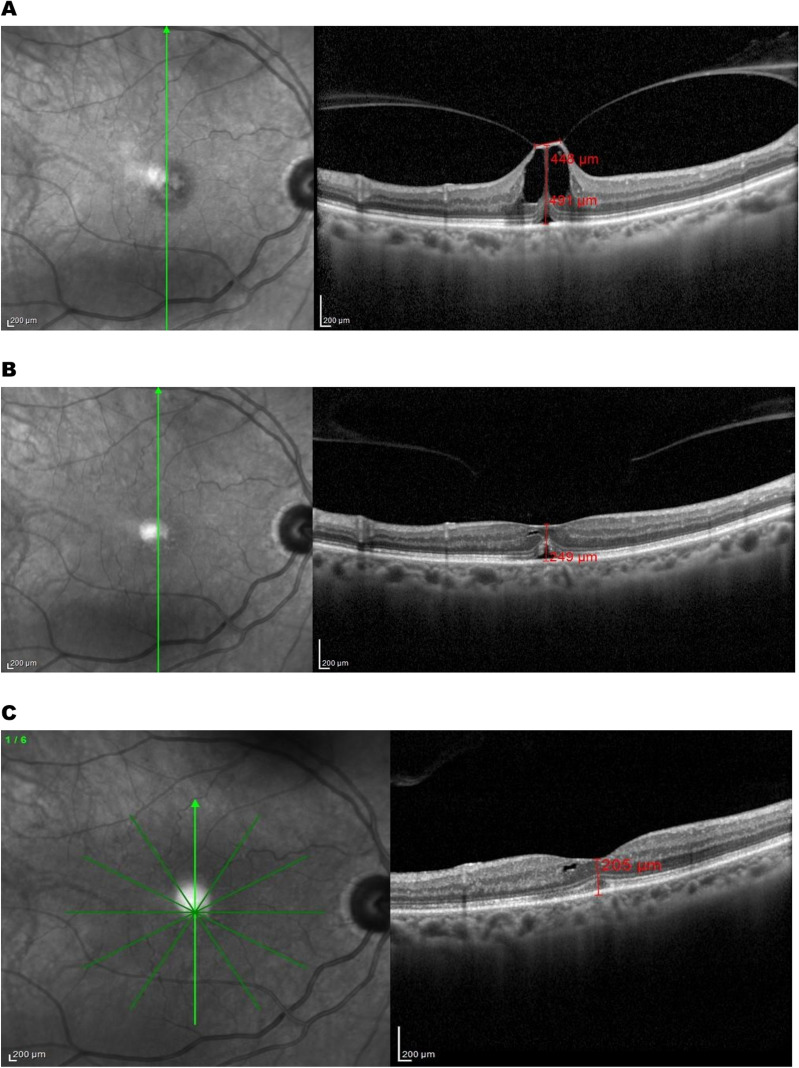
**A** SD-OCT image of isolated focal VMT with CFT 491 µm. **B** Same patient one week after SF6 gas injection showing release of VMT. **C** Restoration of foveal contour after three months of injection

Thirty eyes of 29 patients with symptomatic focal VMT were included. Mean age was 63 years (range 54–74 years; SD ± 5.27). Follow-up period was 3 months. Female patients constituted 72% of the study population. The ratio between phakic and pseudophakic eyes was 2:1. Thirteen eyes (43.3%) had concurrent diabetic maculopathy, 4 eyes (13.3%) had concurrent ERM, one eye had concurrent branch retinal vein occlusion (BRVO) and another one had concurrent dry AMD. Mean preoperative BCVA was 20/125. Mean preoperative CFT was 382µm (range 149-576µm; SD ± 91.88). The mean extent of HVMA was 424.80µm (range 71-853µm; ± SD 200.31). VMT release was documented on SD-OCT at an average of 3 weeks (range, 1–12 weeks) after gas injection. VMT release was achieved in 24 of 30 eyes (80.0% release rate). Figure 1 shows the mean visual acuity at baseline and at the last visit. Mean postoperative BCVA was 20/40. Mean gain of visual acuity was 5 lines. Figure 2 shows the decrease in mean CFT during follow-up compared to baseline. At the last visit mean CFT value was 279 μm (range 145–520 μm and SD ± 132.35), (*P* = 0.000). A statistically significant negative correlation existed between HVMA size in microns and the release of focal VMT. (P value = 0.002) (Table 1). A negative correlation existed between the change of BCVA and the change of CFT over the follow up period r = − 0.7, − 0.5, − 0.7 at 1 week, 1 month, and 3 months, respectively. Gradual improvement of BCVA was accompanied by gradual decrease in CFT (*P* = 0.000). A significant negative correlation existed between the release time and the change of BCVA from the baseline to the first follow up visit (*P* = 0.002), (r = − 0.60). A significant positive correlation existed between the release time and the change of CFT from the baseline to the first follow up visit (*P* = 0.001) (*r* = 0.65), in the sense that eyes with shorter release time had higher BCVA and less CFT at the first follow up. A significant negative correlation existed between the presence of ERM and the release of focal VMT (*P* = 0.003). Figure 3 VMT release occurred in 25% of eyes with ERM and in 88.5% of eyes without ERM. Presence of diabetic maculopathy was associated with a decrease in the final visual outcome in comparison with patients who did not have any diabetic changes (*P* = 0.003). Similarly, diabetic maculopathy was associated with higher mean CFT at the last visit in comparison with patients with no diabetic changes. (*P* = 0.011). As regards the time taken for VMT release (release time), we found that 58.3% of eyes with total release of VMT occurred at the first week of the follow up period. Figures 4 and 5). Isolated focal VMT occurred in 15 out of 30 eyes (50%) included in the study. Subgroup analysis of this cohort revealed that the increase of mean BCVA over the follow up period was highly significant (P = 0.000) and with more improvement at the final visit in comparison with the whole group of 30 eyes. Mean BCVA at the last visit was 20/32 in this subgroup in comparison with 20/40 for the whole group. Mean CMT at the final visit was (230.87 ± 82.76 microns) in this subgroup compared to (279.57 ± 132.35 microns) for the whole group, (*P* = 0.000). The mean time of release of focal VMT was 1.67 weeks (range 1-5 weeks; SD ± 1.37) in the isolated VMT eyes while in eyes with concurrent conditions other than VMT the mean was 4.67 weeks range 1-12 weeks; SD ± 4.46). In eyes in which VMT release did not occur, we did not detect significant relationship as to the change in BCVA (*P* = 0.875) or CFT (*P* = 0.123). Ocular complications developed in one eye after PVL, a retinal break at upper nasal retina associated with vitreous hemorrhage developed after two weeks of injection. Despite release of VMT in this case after one week and improvement of BCVA from 20/200 to 20/32, vision dropped to counting fingers at 50 cm due to hemorrhage. This patient received laser barrage and medical treatment. Vitreous hemorrhage resolved and no retinal detachment developed. The patient regained BCVA of 20/32.

**Table 1:** Relationship between HVMA and release of VMT, Independent t-test

		HVMA(µm)		Test value	*P*-value
		Mean ± SD	Range		
Release	No	642.5 ± 211.29	302–853	3.509	0.002
	Yes	370.38 ± 159.48	71–622

## Discussion

**Fig. 6:**
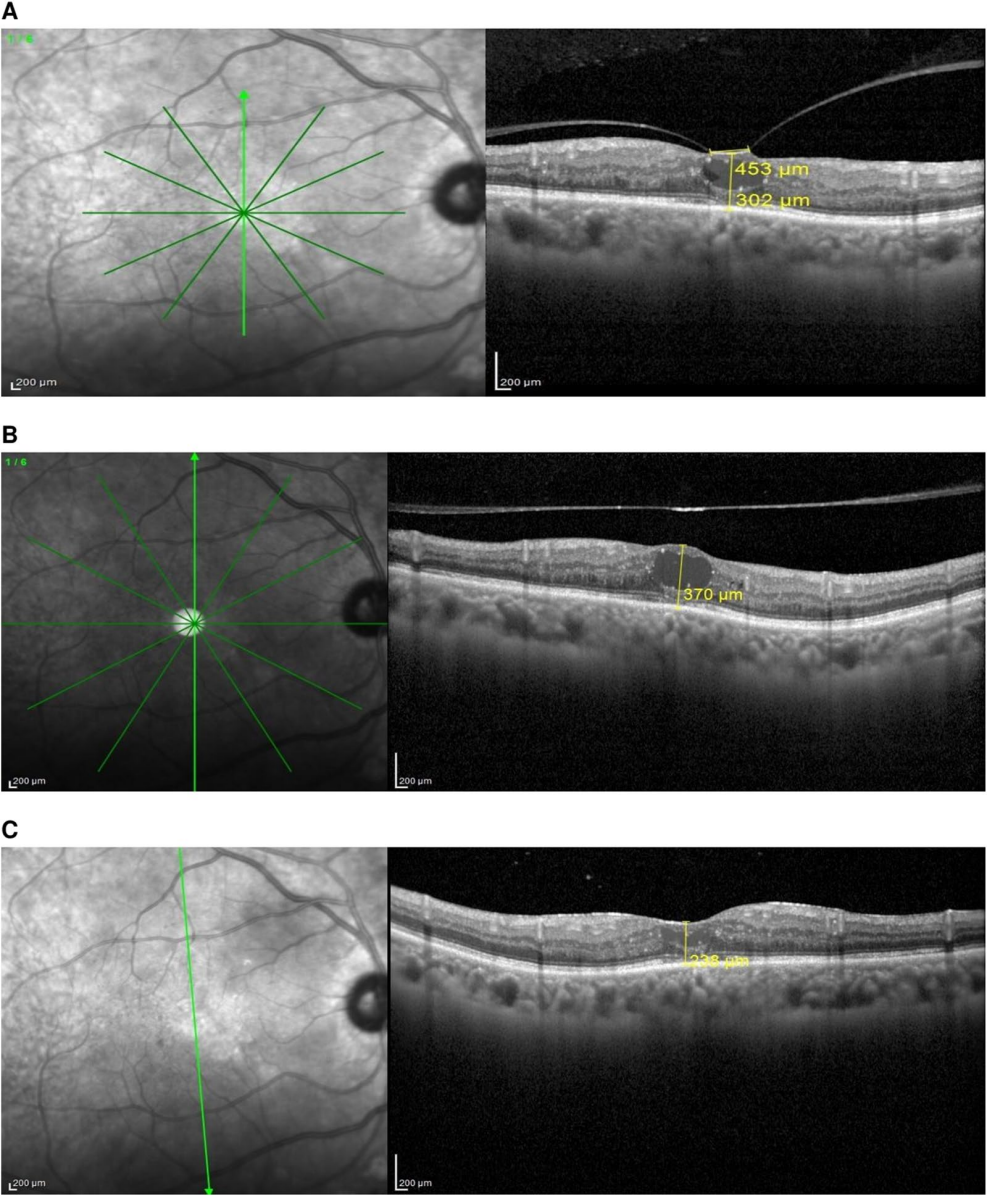
**A** SD-OCT image of a focal VMT with cystoid diabetic macular edema and hard exudates with CFT 302 µm. **B** The same patient after one week of SF6 gas injection with release of VMT. **C** Improvement of the foveal contour after three months of injection and marked decrease of macular edema

Our study assessed PVL as a viable alternative to ocriplasmin and vitrectomy for treating selected cases of symptomatic focal VMT. We had a success rate of 80%, compared to the OASIS trial that used ocriplasmin for symptomatic VMA and reported success rates of 41.7% in the ocriplasmin group versus 6.2% in the placebo group [11]. In comparison to our results, several studies using PVL for focal VMT reported success rates ranging from 95 to 100% [12–14, 14, 15]. On the other hand, Rodrigues et al. [16] and Day et al. [17] reported success rates of 60 and 55.6% respectively. Our results are comparative to Chan et al. [10] and Claus et al. [18] who reported success rates of 84, 85.7 and 84% respectively [10, 18]. Most studies reported the release of VMT at one month, however it could take as long as 9 weeks; therefore, it is prudent to wait for 2 months before switching to an alternative treatment [10, 19]. In our study, the mean duration of VMT release in successful cases was 3 weeks. Almost 70% of eyes with successful VMT release occurred within the first two weeks. In comparison, Claus et al. [18] reported successful VMT release in 52.9% of eyes occurring within the same time period [18]. The mean duration of VMT release reported herein is similar to that reported by Chan et al. [10] who reported mean duration for VMT release of 3 weeks and comparable to Özdemir et al. [15] who reported mean duration for VMT release of 2 weeks [10, 15]. Our study suggests that drinking bird head movements or face-down positioning may accelerate vitreous liquefaction and separation. On the other hand, Rodrigues et al. [16] and Day et al. [17] who had the least success rate of release of VMT did not require their patients to adopt any head positioning after injection [16, 17]. Other studies which had ≥ 80% VMT release rates used face-down posturing or drinking bird head movements [10, 15, 18, 20]. The possible mechanical separation effect provided by these movements may promote VMT release and shorten the time for release. This posture is presumed to roll the gas bubble directly across the macula and the optic nerve to break hyaloidal adhesions and potentiate the release of the VMT. Another important point shown by the present study is that all eyes with successful release of VMT had focal adhesion of ≤ 1500 μm at baseline. This finding is consistent with the report by Rodrigues et al., 2013 who found that broad VMA was considered a poor prognostic factor for the release of VMT after intravitreal gas injection [16]. Our mean HVMA was 424 μm, and that is consistent with literature that suggests that a focal VMA size close or under 500 μm seems to be essential to obtain good results in VMT syndrome [10, 17]. We noticed that isolated focal VMT eyes had better BCVA and CFT at the last follow up visit in comparison to the whole group, The mean of BCVA at the last visit was 20/32 in this isolated VMT group in comparison with 20/40 for the whole group and the mean of CFT at the final visit equal (230.87 ± 82.76) in this isolated group as compared to (279.57 ± 132.35) at the whole group. To our knowledge, this is the first report on this observation. Nevertheless, this finding needs assertion via a prospective comparative stud with larger number of participants. Our results support the hypothesis that lack of diabetic maculopathy is correlated with higher success of VMT release. Accordingly, three eyes with diabetic maculopathy failed to release from 13 eyes have diabetic maculopathy (76.9% success rate), while in non-diabetic patients three eyes failed to release from 17 eyes (82.3% success rate). In addition, there was a trend toward a higher rate of VMT release in eyes without baseline ERM, out of four eyes with ERM just one eye had a release of VMT (25.0% success rate). Our results are in accordance with other studies such as Rodrigues et al. [16] and Chan et al. [10] who also mentioned that success of VMT release was reduced to 25% for eyes with diabetes mellitus and to 50% for eyes with cellophane maculopathy in their studies. [10, 16]. Previous studies on ocriplasmin treatment have reported that younger age (less than 65), lack of cellophane maculopathy, VMT within one disc area, and stage 2 macular hole are strong predictors of success in VMT release [21, 22]. The small sample size in the present study may be the reason for lack of statistical significance of successful VMT release in phakic eyes compared to pseudophakic eyes, despite a larger percentage of phakic eyes (90%) compared with pseudophakic eyes (60%) achieving VMT release. This finding is consistent with Haller et al. [21], who found that presence of the crystalline lens is a predictor of successful VMT release associated with ocriplasmin treatment. Our finding are congruous with (Chan et al. [10]) who reported that 88.4% of phakic eyes achieved VMT release compared of 71.4% of pseudophakic eyes after PVL [10]. In the present study, we used SF6 in all patients because a shorter duration gas may be preferable for PVL in order to defray the possible side effects of a longer acting gas like C3F8, such as the restriction of patient’s daily activities, head positions and mobility. Another important advantage of PVL is that it could be repeated to enhance its success rate although proper case selection is the key to repeat PVL. For example, an eye with a focal VMA that fails to respond to initial PVL may respond favorably to a second intravitreal injection of gas bubble. In our study VMT release was achieved only in 25% of eyes with an ERM. Thus, we do not favor repeat gas injection in these cases. Likewise, eyes with broad and sticky VMA are not good candidates to initiate or repeat PVL. It is noteworthy that PVL may serve as an adjunctive procedure for releasing focal VMT in addition to its potential as a primary procedure. For instance, cases of diabetic macular oedema or any other retinal pathologies that do not respond well to medical treatment due to concurrent focal VMT could benefit from PVL as a less invasive alternative than vitrectomy. Figure 6. For the majority of our cases, anatomical and visual success could be achieved with this low-cost procedure while circumventing the higher expenses and inherent risks of vitrectomy. In our study, VMT release failed to develop in 6 eyes of which 3 eyes had diabetic maculopathy and 2 eyes had ERM and one eye had isolated focal VMT. It is possible that the eyes with diabetic maculopathy and ERM had broader or stronger VMA, which may account for a less robust response to PVL [10]. The last case with isolated focal VMT had HVMA <500 μm and had at the last visit partial release of the central portion of VMT, which suggested that it may have needed longer follow up period or another injection to achieve release of VMT. In the present study, we did not detect any case of uveitis, endophthalmitis, excessive IOP , cataract progression, lenticular dislocation, or zonular dehiscence. In addition, there were no abnormalities noted on SD-OCT after PVL. Limitations of the present study include lack of eyes with VMT concurrent with macular hole, small sample size, lack of concurrent control group . However, only 10.1% of control eyes receiving placebo saline injections had resolved V|MA achieved a PVD in the combined cohorts of the MIVI-TRUST Trial [22], and no more than approximately one third of eyes developed spontaneous VMA/VMT release in two reports on the natural history of VMT without therapeutic intervention [23, 24]. In addition, the development of VMT release shortly after intravitreal gas injection in the majority of the treated eyes in our study is highly suggestive of a causal relationship between PVL and VMT release. Although this study showed that the presence of diabetic maculopathy and thick cellophane maculopathy may reduce the success rates of PVL, the limited sample size associated with both conditions precluded a definitive conclusion. Finally, further prospective studies with longer follow up period are needed to evaluate if there PVL-induced VMT release could develop after three months without further intervention.

## Conclusion

PVL with limited face-down position is a viable option for treating focal VMT with few adverse events. Further studies are needed to evaluate its indications, benefits, and risks.

## Data Availability

The data used in this study are available from the corresponding author on reasonable request.
